# Relationship Between Clinical Symptoms and Skin Autofluorescence in Hemodialysis Patients as a Measure of Advanced Glycation End-Product Accumulation

**DOI:** 10.7759/cureus.27081

**Published:** 2022-07-20

**Authors:** Masahiro Suzuki, Eiji Hanaoka, Yuki Shiko, Yohei Kawasaki, Seiji Ohtori

**Affiliations:** 1 Orthopedic Surgery, Sannou Hospital, Chiba, JPN; 2 Orthopaedic Surgery, Japan Community Health Care Organization Chiba Hospital, Chiba, JPN; 3 Biostatistics Section, Clinical Research Center, Chiba University Hospital, Chiba, JPN; 4 Clinical Research Center, Chiba University Hospital, Chiba, JPN; 5 Orthopaedic Surgery, Chiba University, Chiba, JPN

**Keywords:** visual analog scale, japanese orthopaedic association back pain evaluation questionnaire, hemodialysis, skin autofluorescence, advanced glycation end-products

## Abstract

Background

The purpose of this study was to investigate the relationship between skin autofluorescence (SAF), as a measure of advanced glycation end-product (AGE) accumulation and osteoporosis and clinical symptoms in hemodialysis patients.

Methodology

The study participants were 156 hemodialysis patients (97 males, 59 females, mean = 66.9 years, range = 25-92 years) who visited our hospital between October 2019 and March 2020. The average dialysis period was 10.4 years (range = 1-40 years). Age, years of dialysis, bone mineral density, bone metabolism markers (Ca, P, intact parathyroid hormone, total N-terminal propeptide of type 1 collagen, tartrate-resistant acid phosphatase-5b), clinical symptoms, and SAF were evaluated. Clinical symptoms were evaluated using the visual analog scale (VAS) score for low back pain (LBP) and leg pain ranging from 10 mm (extreme amount of pain) to 0 mm (no pain); the Japanese Orthopaedic Association Back Pain Evaluation Questionnaire (JOABPEQ; 0-100 points); and the Roland-Morris Disability Questionnaire (RDQ; 0-24 points). We calculated Pearson correlation coefficients to assess the correlation of SAF with age, years of hemodialysis, bone density, bone metabolism markers, clinical symptoms, and biochemical markers.

Results

The SAF of dialysis patients averaged 4.11, higher than previous reports for non-dialysis patients. Age (r = 0.435, p = 0.0001) was moderately positively correlated and hemodialysis period (r = 0.214, p = 0.00907) was weakly positively correlated with SAF. Among the clinical symptoms measured by the JOABPEQ, social life dysfunction (r = -0.257, p = 0.0108) had a weak negative correlation with SAF.

Conclusions

The level of AGEs implied by SAF was elevated in hemodialysis patients. SAF correlated with social life disorders, suggesting that SAF may be involved in disorders of activities of daily living in hemodialysis dialysis patients.

## Introduction

In 2020, the number of hemodialysis patients in Japan exceeded 340,000 and is expected to continue to increase every year. Furthermore, the number of patients with a hemodialysis history of more than 40 years is increasing due to advances in dialysis technology and systemic management. In long-term hemodialysis patients, spondylosis develops from dialysis-related amyloidosis, age-related spondylosis, and renal osteodystrophy. In dialysis-related amyloidosis, bone and joint lesions are the main cause of decreased quality of life because amyloid fibrils (Aβ2M), which are precursor proteins of β2-microglobulin (β2-MG) that increase in the blood, are deposited in the extracellular tissues of bones and joints [1-4].

Serum β2-MG does not necessarily correlate with the incidence of dialysis-related amyloidosis, and associations such as dialysis history, aging, advanced glycation end products (AGEs), chronic inflammation, and oxidative stress have been reported. In 1984, destructive spondyloarthropathy (DSA) of the cervical spine in long-term hemodialysis patients was first described by Kunz et al. [5]. The radiological features of DSA include narrowing of the intervertebral disc space, bone erosions, and cyst formation in the adjacent vertebral endplates.

AGEs are a diverse class of molecules produced by non-enzymatic saccharification of proteins, lipids, and nucleic acids [6]. They are uremic toxins, and their levels are significantly elevated in patients with chronic kidney disease (CKD), especially those on dialysis, due to the increased production, impaired excretion, and inefficient removal of these toxins [7,8]. AGEs are rapidly produced via either endogenous sources such as hyperglycemia and oxidative stress or exogenous sources such as cigarette smoking and diet. AGEs form crosslinks with tissue proteins and interact with specific AGE receptors to promote systemic inflammation, thereby exacerbating tissue damage [9]. These AGE crosslinks impair the mechanical and biological properties of bone [10,11].

A new non-invasive device has been developed to measure AGE levels via quantification of skin autofluorescence (SAF), which was recently proposed as a marker of AGE accumulation in the skin [12]. Eguchi et al. demonstrated that women with osteoporotic vertebral fractures have elevated SAF (2.38 vs. 2.25, p = 0.0002) compared with women without fractures [13]. Umimura et al. reported an association between SAF and chronic low back pain (LBP). Thus, SAF may be an indicator of chronic LBP [14].

SAF intensity increases over time in most people on dialysis. Independent predictors of a higher SAF intensity are malnutrition development/prevalence, hemodialysis as the first dialysis modality, and current smoking status [15]. However, there are no reports on the association between SAF and clinical symptoms such as LBP in hemodialysis patients. Assessing the severity of spondylosis in hemodialysis patients is important and SAF may be a biomarker. The purpose of this study was to investigate the relationship between SAF (as a measure of AGE accumulation) and osteoporosis and clinical symptoms in hemodialysis patients.

## Materials and methods

Study participants

Informed consent was obtained from all participants prior to study initiation. The study protocol was approved by the Ethical Review Committee (approval number: 2428).

Of the 200 hemodialysis patients who visited our hospital from October 2019 to March 2020, 156 patients cooperated with this survey and underwent bone density and blood sampling (97 males, 59 females, mean = 66.9 years, range = 25-92 years). They had been on hemodialysis for an average of 10.4 years (range = 1-40 years). Exclusion criteria were a history of spinal surgery and neuromuscular diseases such as Parkinson’s disease.

Dual-energy X-ray absorptiometry

The bone mineral density (BMD) of the left proximal femur and lumbar spine (L2-L4) was measured by dual-energy X-ray absorptiometry (Lunar Prodigy, GE Healthcare, WI, USA). The Young Adult Mean (YAM) (%) of BMD in the lumbar spine and femoral neck were used for analysis.

Measurement of SAF

SAF was measured using DiagnOptics Technologies BV (Groningen, Netherlands), a non-invasive automated device that measures the characteristic fluorescence properties of certain AGEs to estimate the degree of AGE accumulation in the skin. AGEs were measured twice on the same day in the forearm on the opposite side of the vascular-access side of the hemodialysis patient, and the average value was used. The technical details have been described previously. SAF is expressed in arbitrary units. SAF measurements were conducted at room temperature on the ventral forearm of the patient while in the sitting position.

We evaluated age, years of hemodialysis, bone mineral density, bone metabolism markers (Ca, P, intact parathyroid hormone (PTH), total N-terminal propeptide of type 1 collagen (P1NP), tartrate-resistant acid phosphatase-5b (TRACP-5b), β2-MG), clinical symptoms, and SAF. Clinical symptoms were evaluated using the visual analog scale (VAS) score for LBP, leg pain, and leg numbness, ranging from 100 mm (extreme amount of pain) to 0 mm (no pain); the Japanese Orthopaedic Association Back Pain Evaluation Questionnaire (JOABPEQ; 0-100 points); and the Roland-Morris Disability Questionnaire (RDQ; 0-24 points).

The JOABPEQ includes 25 questions based on RDQ and Short-Form 36 (SF-36) pertaining to the following five domains: pain-related disorders, lumbar spine dysfunction, gait disturbance, social life dysfunction, and psychological disorders. The score for each domain was calculated according to the official guidelines (A3) and ranged from 0 to 100 points, with 0 representing the worst and 100 the best clinical condition of the patient. The normal RDQ is zero points, with the total number of items checked from a minimum of 0 to a maximum of 24.

Pearson’s correlation coefficients were calculated to assess the correlation of SAF with age, hemodialysis period, bone density, bone metabolism markers, clinical symptoms, and biochemical markers.

All data are expressed as the mean ± standard deviation (SD). Statistical analysis was performed with SAS software version 9.4 (SAS Institute, Cary, NC, USA). P-values of <0.05 were considered statistically significant.

## Results

Table 1 shows the physical findings, bone density, bone metabolism markers, clinical symptoms, and SAF in hemodialysis patients.

**Table 1:** Characteristics of study participants. BMD: bone mineral density; PTH: parathyroid hormone; P1NP: N-terminal propeptide of type 1 collagen; TRACP-5b: tartrate-resistant acid phosphatase-5b; VAS: visual analog scale; JOABPEQ: Japanese Orthopaedic Association Back Pain Evaluation Questionnaire; RDQ: Roland-Morris Disability Questionnaire; SAF: skin autofluorescence

	Items
Physical examination	
Number of subjects	150
Age (years)	66.91 ± 13.63
Hemodialysis period (years)	10.43 ± 8.15
Number of patients with diabetes mellitus (%)	53 (35.3)
BMD (Young Adult Mean)	
Lumbar (%）	96.19 ± 20.93
Femoral (%）	78.44 ± 16.44
Serum testing checklist	
K (mEq/L)	5.03 ± 1.25
Ca (mEq/L)	8.47 ± 0.61
P (mEq/L)	5.39 ± 1.47
Intact PTH (pg/mL)	172.07 ± 117.23
Alb (g/dl)	3.44 ± 0.39
Β2 microglobulin (mg/L)	28.45 ± 6.46
Total P1NP (ng/mL)	421.35 ± 275.44
TRACP-5b (mU/dL)	646.26 ± 415.71
Clinical symptoms	
VAS	
Low back pain (mm)	28.0 ± 30.8
Leg pain (mm)	16.6 ± 25.3
Leg numbness (mm)	11.5 ± 23.1
JOABPEQ	
Pain-related disorders	71.49 ± 35.0
Lumbar spine dysfunction	76.28 ± 29.19
Social life dysfunction	65.79 ± 27.43
Gait disturbance	68.28 ± 35.64
Psychological disorders	54.51 ± 17.07
RDQ	4.85 ± 5.85
SAF (AU)	4.11 ± 0.95

**Figure 1:**
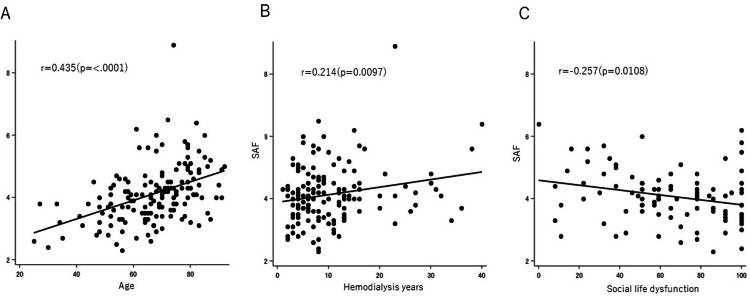
Correlation of SAF with age (A), hemodialysis period (B), and social life dysfunction (C). SAF: skin autofluorescence

Age (r = 0.435, p = 0.0001) was moderately positively correlated and hemodialysis period (r = 0.214, p = 0.0097) was weakly positively correlated with SAF. Among clinical symptoms, social life dysfunction assessed by the JOABPEQ (r = -0.257, p = 0.0108) had a weak negative correlation with SAF (Figure 1).

No correlation was found between SAF and bone mineral density, bone metabolism markers, or clinical symptoms other than JOABPEQ social life dysfunction. No correlation was also found between SAF and β2MG levels (r = 0.077, p = 0.38).

## Discussion

In this study, we investigated the relationship between SAF (as a measure of AGE accumulation) and osteoporosis and clinical symptoms in hemodialysis patients. The SAF in hemodialysis patients (mean = 66.9 years) was 4.11, which was higher than the previously reported 2.25 in non-dialysis patients (mean = 72.0 years), despite being younger [13]. SAF showed a moderate positive correlation with age and a weak positive correlation with the hemodialysis period. Among JOABPEQ domains, social life dysfunction was weakly and negatively correlated with SAF.

Chikuda et al. used a large database to compare the perioperative risk of dialysis patients who underwent spinal surgery with non-dialysis patients and found that the incidence of postoperative complications increased by 60% and the risk of death by 10 times (odds ratio = 9.81) in the dialysis group [16]. Based on plain X-ray images, Yamamoto et al. that 20% of hemodialysis patients develop DSA. The duration of hemodialysis and the age at which hemodialysis is started are risk factors for developing DSA; however, they found no association between serum β2-MG levels and the development of DSA [17].

AGEs, a representative uremic toxin, have been shown to cause a variety of CKD-related complications. Various CKD-related pathological conditions, such as chronic inflammation, are associated with the onset and progression of sarcopenia. In hemodialysis patients, serum AGE levels increase significantly with frailty and are inversely proportional to physical performance and activity [18].

Eguchi et al. showed that sarcopenia is associated with spinal deformations and increased LBP [19]. High serum levels of pentosidine, a representative product of AGEs, are associated with the severity of spinal malalignment in older women, suggesting that high levels of AGEs are a potential biomarker of the progression of lumbar scoliosis and kyphotic deformity [20]. Umimura et al. demonstrated that AGE levels, as determined by SAF measurements, were significantly higher in patients with chronic LBP, suggesting that SAF can be used as an indicator of chronic LBP [14].

Regarding the relationship between serum pentosidine and SAF, Hashimoto et al. [21] found no correlation between SAF and serum pentosidine in type 2 diabetes patients. Fokkens et al. [22] showed that serum pentosidine concentrations were significantly increased but SAF was not increased in retinal detachment patients with type 2 diabetes. On the other hand, several reports showed that serum pentosidine and SAF significantly increased in patients with type 2 diabetes [23,24] and hemodialysis [12]. The dynamics of SAF and serum pentosidine in diabetic and dialysis patients need to be investigated.

In this study, increased SAF (as a measure of AGE accumulation) was associated with social life dysfunction in hemodialysis patients. Activities of daily living (ADL) may decline with dialysis, spondylosis, and spinal deformity in hemodialysis patients. Therefore, SAF may be involved in disorders in ADL in hemodialysis dialysis patients.

This study has several limitations. First, we did not measure serum pentosidine in dialysis patients. Second, the presence or absence of spondylosis, spinal fractures, and pyogenic spondylitis was not confirmed using plain X-ray images. LBP can be caused by multiple factors, including spondylosis and spinal fractures, and the heterogeneity of these patients can bias the outcomes. Finally, this study does not consider confounding factors related to age, dialysis duration, or dysfunction. In the future, matching will need to be considered to eliminate the effects of confounding factors.

## Conclusions

The level of AGEs implied by SAF in hemodialysis patients was higher than that in non-dialysis patients. SAF was positively correlated with age and the hemodialysis period. SAF was weakly correlated with social life disorders. SAF correlated with social life disorders, suggesting that SAF may be involved in disorders of ADL in hemodialysis dialysis patients.
